# Accurate Crack Detection Based on Distributed Deep Learning for IoT Environment

**DOI:** 10.3390/s23020858

**Published:** 2023-01-11

**Authors:** Youngpil Kim, Shinuk Yi, Hyunho Ahn, Cheol-Ho Hong

**Affiliations:** 1 Department of Information and Telecommunication Engineering, Incheon National University, 119, Academy-ro, Yeonsu-gu, Incheon 22012, Republic of Korea; 2 Metaverse World Co., 134, Teheran-ro, Gangnam-gu, Seoul 06235, Republic of Korea; 3 School of Electrical and Electronics Engineering, Chung-Ang University, 84 Heukseok-ro, Dongjak-gu, Seoul 06974, Republic of Korea

**Keywords:** crack detection, edge computing, U-Net, Efficient-Net

## Abstract

Defects or cracks in roads, building walls, floors, and product surfaces can degrade the completeness of the product and become an impediment to quality control. Machine learning can be a solution for detecting defects effectively without human experts; however, the low-power computing device cannot afford that. In this paper, we suggest a crack detection system accelerated by edge computing. Our system consists of two: Rsef and Rsef-Edge. Rsef is a real-time segmentation method based on effective feature extraction that can perform crack image segmentation by optimizing conventional deep learning models. Then, we construct the edge-based system, named Rsef-Edge, to significantly decrease the inference time of Rsef, even in low-power IoT devices. As a result, we show both a fast inference time and good accuracy even in a low-powered computing environment.

## 1. Introduction

Cracks in roads, building walls, floors, and product surfaces can degrade the safety of the infrastructure or the quality of the product. Fine cracks that are difficult to detect through human eyes may require the inspection of experienced experts with a sophisticated inspection tool. However, employing experienced experts is expensive, and there is also a possibility of mistakes by the experts. Also, there are cases in which access itself is difficult, such as the inspection of cracks in the bridge. Several studies on bridge crack detection based on unmanned aerial vehicles (UAV) have been recently proposed [1,2,3]. The UAV-based detection system captures images of the bridge surface through a camera and transmits them to a cloud server to determine whether cracks are detected. Scalability problems in performance can arise when a large number of devices are deployed for collecting and processing data. Edge computing can reduce the computing burden concentrated on the cloud server to the edge server by lowering it to a manageable level [3]. In this case, edge IoT devices such as UAVs can focus on data collection, increasing their efficiency.

Advances in deep learning technologies play a significant role in image processing for crack detection. As the deep learning structure becomes richer and deeper, various feature expressions and feature learning have become possible. For example, VGG [4], GoogleNet [5], ResNet [6], and AlexNet [7] showed significant results in image feature ex- traction. However, many parameters must be sufficiently processed to reflect the extracted features. The problem is that the edge IoT device lacks the computing power to process the extracted parameters. Compared to cloud servers, poor computation performance and slow network speed can degrade the efficiency of crack detection. In this context, a crack detection technique that can operate in UAV devices by reducing the number of parameters in the backbone network for feature extraction has been suggested. However, there are no evaluation results of the actual edge environment, and the use case of edge cloud servers is not provided.

In this paper, we design and implement an edge-based crack detection mechanism based on deep learning. Our suggestion consists of two parts: a crack detection model part and an edge computing part. We develop a crack detection model called Real-Time Segmentation using Effective Feature Extraction (Rsef). Rsef is a semantic segmentation model for crack detection based on EfficientNet and U-Net, can operate with a small dataset, and shows a fast inference time. We also construct Rsef-Edge, the edge-based system that supports Rsef on a low-power IoT device. Rsef-Edge provides a computation offloading functionality with distributed inference. Rsef-Edge exploits the computation resources of both an IoT device and the edge server with a powerful GPU for rapidly detecting cracks in images. Thus, Rsef-Edge can be applied to a scenario where the user has a performance- limited IoT device and requires fast inference latency. For evaluation of Rsef-Edge, we use NVIDIA Jetson TX2, which is one of the industrial IoT computers. From the results, Rsef-Edge can improve the inference latency by up to 17.40 times without an accuracy drop.

This paper consists of the following sections. Section 2 explains the related work. In Section 3, we describe background including issues about machine-learning-based crack detection and edge computing. Section 4 explains our suggestions, architecture, and imple- mentation details. Section 5 evaluates the experimental results, and finally, we conclude in Section 6.

## 2. Related Work

Studies applying machine learning to concrete crack detection have been steady since the late 1990s, and deep-learning-based methods have been proposed since 2017 [8]. Deep-learning-based methods can be divided into classification, object detection, and segmentation. In this paper, we adopt a segmentation-based method. Segmentation produces pixel-level predictions of cracks in the image. That is, the existence of a crack can be predicted by overlapping the original image and the crack detection result. Regarding segmentation for crack detection, the study [8] classified five approaches as follows. The first is an encoder–decoder-based model, to which many studies [9,10,11,12,13] belong. In the encoder–decoder model, fully convolutional networks (FCN) [14] and SegNet [15] were used. For generalized semantic segmentation, researchers added hidden layers to avoid the loss of spatial information due to downsampling. As approaches to which this is applied, studies using a model using crack segmentation without a pooling layer are the second approach [16,17,18]. Third, the CNN model is used to combine classification tasks with other proposed techniques. Other than that, there are studies using RNN [18] or GAN [19] for crack segmentation.

On the other hand, there are not many studies using crack detection for edge comput- ing. In the study of [3], to detect bridge cracks, UAV technology is applied to connect to the cloud server, and crack detection is performed. The authors suggested a feature map fusion to reduce the load on the cloud server caused by the increase in the number of UAVs.

As deep learning technology advances, there is an active trend to study pixel-based segmentation in traditional crack classification. Computation requirements such as real-time inference time and scalability have increased; the need for edge computing has increased. We follow such latest trends. The difference from the recent study [3] is that our method is more straightforward because it does not require the creation of additional low-level feature maps.

## 3. Background

### 3.1. Image Data Generator

Image data generator (IDG) [20] is a data augmentation library that Keras provides for enhancing training performance. Data augmentation has been used for data transformation to improve data diversity in the computer vision area. IDG in the Keras package consists of rotation_range (image rotation value), shear_range (image tilt), zoom_range (left/right movement), width_shift_range (up/down movement), height_shift_range (image hori- zontal flip), horizontal_flip (image horizontal flip), vertical_flip (image vertical flip), and other variations are available. When IDG is used, training performance can be improved by combining various transformations according to each class characteristic.

### 3.2. Albumentations: Fast and Flexible Image Augmentations

Image augmentation is intensively used in the environment where it suffers a lack of images problem because it can generate additional image data using various image trans- formations. Especially, biological and medical images are well-known examples [21,22]. In this environment, a variety of sample generation and the speed of transformation can be beneficial for providing more various data combinations. Simple data augmentations such as IDG are often limited to simple transformations such as flipping, rotating, scaling, and copping. Albumentation [23] can additionally use a variety of additional variations such as MultiplicativeNoise, ToSepia, JpegCompression, ChannelDropout, ChannelShuffle, Cutout, InvertImg, RandomGridShuffle, Blur, and so on. In particular, albumentation has excellent performance improvement of ElasticTransform in low-level images [22]. The speed of transformation in albumentation is known as quick [23].

### 3.3. Skip Connection

The detailed pixel information of an image in the model can disappear [14] during the sampling process, such as downsampling and upsampling. This is a big problem in segmentation. Spatial information can be recovered [24] using a skip connection from the encoder to the decoder. Skip connections were first introduced in FCN for semantic segmentation [14]. With skip connection, better prediction is possible by reflecting precise pixel information [14]. The skip connection is useful in deep neural network architectures such as residual networks (ResNet) [6] and dense networks [25,26]. It is also beneficial to enhance gradient flow and improve the performance of overall classification networks [6].

### 3.4. EfficientNet

EfficientNet [27] is a mobile-size baseline compound model inspired by the study [28]. EfficientNet can evaluate the model scales using existing ConvNets. The authors suggested various types of EfficientNets (B0 to B7) according to various compound coefficients. In the paper, the authors showed that EfficientNet could make 8.4× smaller and 6.1× faster on inference than existing ConvNet by optimizing accuracy and FLOPS.

### 3.5. U-Net

U-Net [22] is a fully convolutional neural network developed for biomedical image segmentation. U-Net has a symmetrically shaped network consisting of two paths: con- tracting and expansive path. The contracting path is based on a typical convolutional network for downsampling the feature map. The expansive path consists of multiple steps of upsampling with concatenation with the corresponding feature map from the contracting path. A network in U-Net is trained end-to-end from a few images and is fast. Thus, it is beneficial to microscopy images such as cells.

### 3.6. Edge Computing

In the study [29,30], the authors describe “edge computing refers to the enabling technologies allowing computation to be performed at the edge of the network, on downstream data on behalf of cloud services and upstream data on behalf of IoT services.” In other words, IBM (https://www.ibm.com/cloud/what-is-edge-computing, accessed on 20 November 2022) defines edge computing as a distributed computing framework that brings high-performance or computation-intensive applications closer to IoT devices or edge servers. This proximity can benefit from faster response times and better bandwidth availability.

## 4. Our Suggestion

We design and implement a live crack detection mechanism based on machine learning. Our suggestion consists of two parts: a machine learning part and an edge computing part. A machine learning part describes how we build our model for crack detection with architecture and block structure for improving accuracy. An edge computing part explains how we design and implement edge cloud to enhance the inference time.

### 4.1. Rsef Process

**Figure 1:**
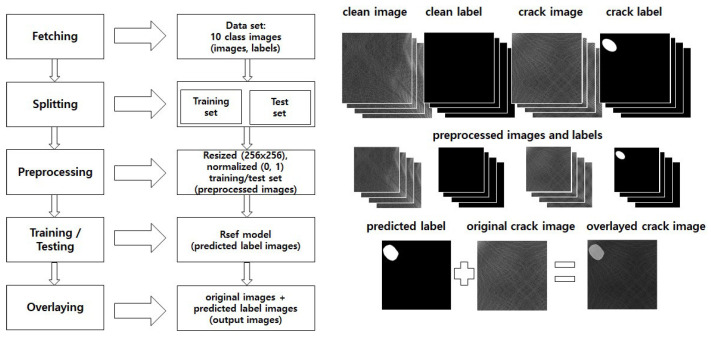
Overall process of Rsef.

Figure 1 shows the overall process of Rsef with operations and outputs.

Crack detection in Rsef consists of five stages: fetching, splitting, preprocessing, training/testing, and overlaying. In the fetching stage, a dataset that consists of various texture images is stored in the storage device. We use a dataset—DAGM 2007 [31], which is a synthetic benchmark for defect detection of texture surface images and has ten classes of texture groups. The DAGM 2007 dataset is widely employed by crack detection studies and can be used for segmentation. Images in the dataset consist of two types: raw images and labels. Raw images contain diverse textures and are categorized into clean and crack images. Crack images are images with cracks or defects, and the others are clean images. Thus, the purpose of segmentation is to detect the above images correctly. Labels indicate ground truths for the segmentation; that is, clean images have a single black image and crack images have white regions per image. We consider that a white region should cover the crack in the right place if there is a crack in the image; thus, it is a segmentation issue (where is the crack region if it exists?). Next, in the splitting stage, we split the images in the dataset into two subsets: training and test set. Each subset goes through the preprocessing stage, where the images are adjusted to an easy-to-handle and efficient form for the neural network. Specifically, all images are resized to 256 × 256 pixel-sized images, and the feature values are normalized to numbers between 0 to 1 for feature scaling. The results are preprocessed images and normalized feature values. The images and values are inputs for a Rsef model, which is generated in the training stage and used in the testing stage. The Rsef model predicts the crack pixel positions corresponding to white regions, and it is used for producing a predicted label. The details of the Rsef model are described in Section 4.2. The overlaying stage, our last stage, produces an overlayed crack image that combines the predicted label and the corresponding original crack image. The predicted label has the same size as the original image because the image size is adjusted to the original size when yielding an output image.

Rsef implements feature extraction based on an Encoder–Decoder structure. As an encoder and decoder, we adopt EfficientNet and U-Net, respectively. First, the encoder re- duces the feature map by downsampling the image progressively to capture the high-level details of the original image. Unlike CNN-based encoders, EfficientNet adopts MBConv with the squeeze and excitation optimization as the basic building block. MBConv [32] proposed Depthwise Separable Convolution as a structure for running CNN models on mobile devices. In MBConvV1, the number of parameters and dimensions are reduced by splitting and applying convolution per channel and combining the split channels into one. In MBConv2, an inverted residual structure with a linear bottleneck was proposed to reduce the amount of computation. Squeeze-and-excitation [33] is an optimizing technique for adaptively recalibrating channelwise feature responses by explicitly modeling interde- pendencies between channels. Squeeze-and-excitation can be attached to existing networks. In this way, the EfficientNet structure gradually reduces the input resolution of the image to produce the final feature map. Second, the decoder in Rsef rebuilds the segmentation map from the reduced feature map to recover spatial information by upsampling the feature map of the encoder. Rsef uses the U-Net structure for this decoder, and features can be recovered from optimized feature maps produced by EfficientNet.

### 4.2. Rsef Model

**Figure 2:**
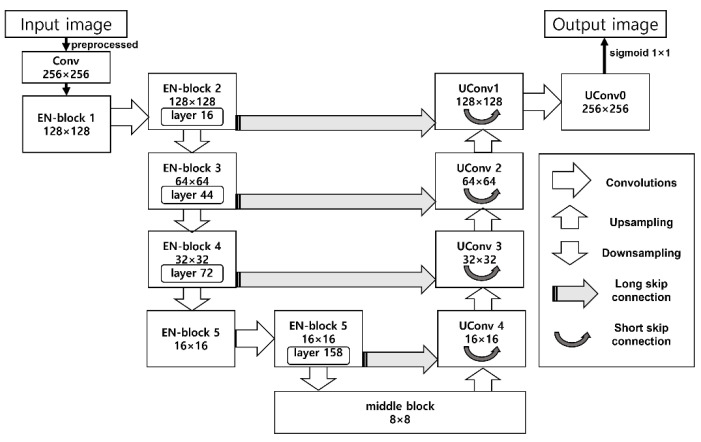
Architecture of Rsef.

Now, we explain the architecture of the Rsef model in order to explain how we predict the crack pixel position. Figure 2 shows the architecture of Rsef.

Rsef is based on U-Net, but we replaced the contracting path (original encoding part of U-Net) with lightweight EfficientNet [27] for lower latency. Contracting path of original U-net is a typical CNN, so it is not specialized for live crack detection in an IoT environment. In EfficientNet, a base convolution network is represented as ConvNet [27]. ConvNet *i* can be defined as a function: *Y_i_* = *F_i_*(*X_i_*), where *F_i_* is the operator, *Y_i_* is output tensor, *X_i_* is input tensor, with tensor shape *<H_i_*, *W_i_*, *C_i_>*, where *H_i_* and *W_i_* are spatial dimension and *C_i_* is the channel dimension. ConvNet *N* is a list of composed layers and defined as:

*N* = *⊙*_*i*=1…*s*_ (*F^Li^* (*X*_<*H_i_*, *W_i_*, *C_i_*>_)) (1)

where *⊙*(*·*) is a list of composed layers partitioned into multiple stages and all layers in each state share the same architecture; *F^Li^* denotes layer *F_i_* is repeated *L_i_* times in stage *i*, <*H_i_*, *W_i_*, *C_i_*> denotes the shape of input tensor *X* of layer *i*.

To find optimized network width (*w*), depth (*d*), and resolution (*r*), EfficientNet defines Scalable ConvNet *N* as follows.

(2) $$N(d,w,r)\;=\;{⊙}_{i\;=\;1\ldots s}\;({\hat{F_{i}}}^{d\hat{L_{i}}}(X_{<r\;\hat{H_{i}},\;r\;\hat{W_{i}},\;w\;\hat{C_{i}}>}))$$

where $\hat{F_{i}}$, $\hat{L_{i}}$, $\hat{H_{i}}$, $\hat{W_{i}}$, $\hat{C_{i}}$ are predefined parameters in the base network, *d*, *w*, *r* are optimized coefficients in maximizing accuracy with limitations of target memory and target flops by a compound scaling method, which uses a compound coefficient to uniformly scales network width, depth, and resolution in a principled way [27].

EfficientNet provides a flexible and efficient compound model structure for validating various model parameters. The basic building block of EfficientNet is MBConv with the squeeze and excitation optimization, and Rsef also uses an MBConv-based structure denoted as EN-block in Figure 2. An EN-block consists of multiple layers of MBConv operators with various kernel sizes (3 × 3 or 5 × 5), and its resolution and channels can be varied as input data. Table 1 indicates the structure of EN-Block of Rsef.

EfficientNet pursues the optimal model shape (depth, width, and resolution) under given target memory and FLOPS. According to the EfficientNet study [27], a compound scaling method is introduced to find such a model shape, which uses a uniform coefficient that has an exponential correlation with the shape. In this study [27], eight model types of EfficientNet are presented from B0 (lightweight) to B7 (heavy). Rsef adopts the lightweight B0 model with an accuracy of 93.2%; the heavy B7 achieves the best accuracy of 97.1%. The number of parameters of B0 is the lowest (5.3M) among all EfficientNet model types. Therefore, B0 is suitable for live crack detection in the performance-limited IoT environ- ment. We adjust various model parameter values on EfficientNet-B0 and find our model settings as denoted in Table 1. Building blocks in the encoder part of Rsef are a total of 7: a convolutional block and 6 MBConvs. An input image data through preprocessing transforms to 0-to-1 normalized model values with 256 × 256 pixel size. Then, through up- sampling, the feature data are extracted and abstracted in the Rsef model. Rsef model stores highly abstracted feature data in the middle block. The middle block is an intermediate block for downsampling. In the downsampling path, Rsef amplifies and extends extracted features based on the U-Net decoder, that is, the decoder part. We use a skip connection to resolve feature vanishing issues in the decoder. We use two types of skip connections: short and long skip connections. The short skip connection is used for mitigating the vanishing gradient problem in each upsampling block (UConv 1,2,3,4). The long skip connection is used to reinforce features from each EN-block in the downsampling path to each UConv block in the upsampling path to recover spatial information lost during downsampling. In the last steps, the output image is generated by applying sigmoid 1 × 1 into aggregated the UConv0 block to predict whether each pixel belongs to a crack pixel.

### 4.3. Edge Based System Support for Rsef

We also construct Rsef-Edge, which is an edge-based system support for Rsef on low-power IoT device. Rsef-Edge provides a computation offloading functionality. Modern CNN models demand considerable computation resources for inference as the structure of the algorithms has become deeper. Recent powerful GPUs are required to process such deep learning workloads. However, low-power IoT devices have only ARM CPUs or performance-limited GPUs. Therefore, running the Rsef model in low-power IoT devices would result in unacceptable inference time.

Rsef-Edge proposed in this paper is a framework to accelerate crack detection inference by applying a distributed inference technique. Rsef-Edge slices the Rsef model into two. Each piece is deployed in the low-power IoT device and the edge server, respectively. The IoT device then executes the early layers of the Rsef model, and the edge server with powerful GPUs performs the remaining layers. This distributed inference boosts the speed of crack detection inference significantly without having GPUs in the IoT device.

For the slicing of the model, we have profiled the Rsef model to find appropriate split points. As shown in Figure 2, the Rsef model has certain blocks that have skip connections, which allow each block to connect to a remote block additionally. In Rsef-Edge, the blocks that have skip connections are not selected as a slicing point for distributed inference because transferring skip connection data additionally to the edge server leads to network overhead. As shown in the figure, the output of EN-block 2 has a skip connection to UConv1, and therefore the layers in EN-block 1 and 2 can be a split point. However, we exclude the layers in EN-block 1 in order to enhance privacy. EN-block 1 is an input layer that performs preprocessing and resizing. Therefore, the output of EN-block 1 maintains the original image, and when this image is transferred to the edge server, privacy cannot be preserved. EN-block 2 has convolutional layers, so it is difficult to infer the original image based on the processed image in EN-block 2.

**Figure 3:**
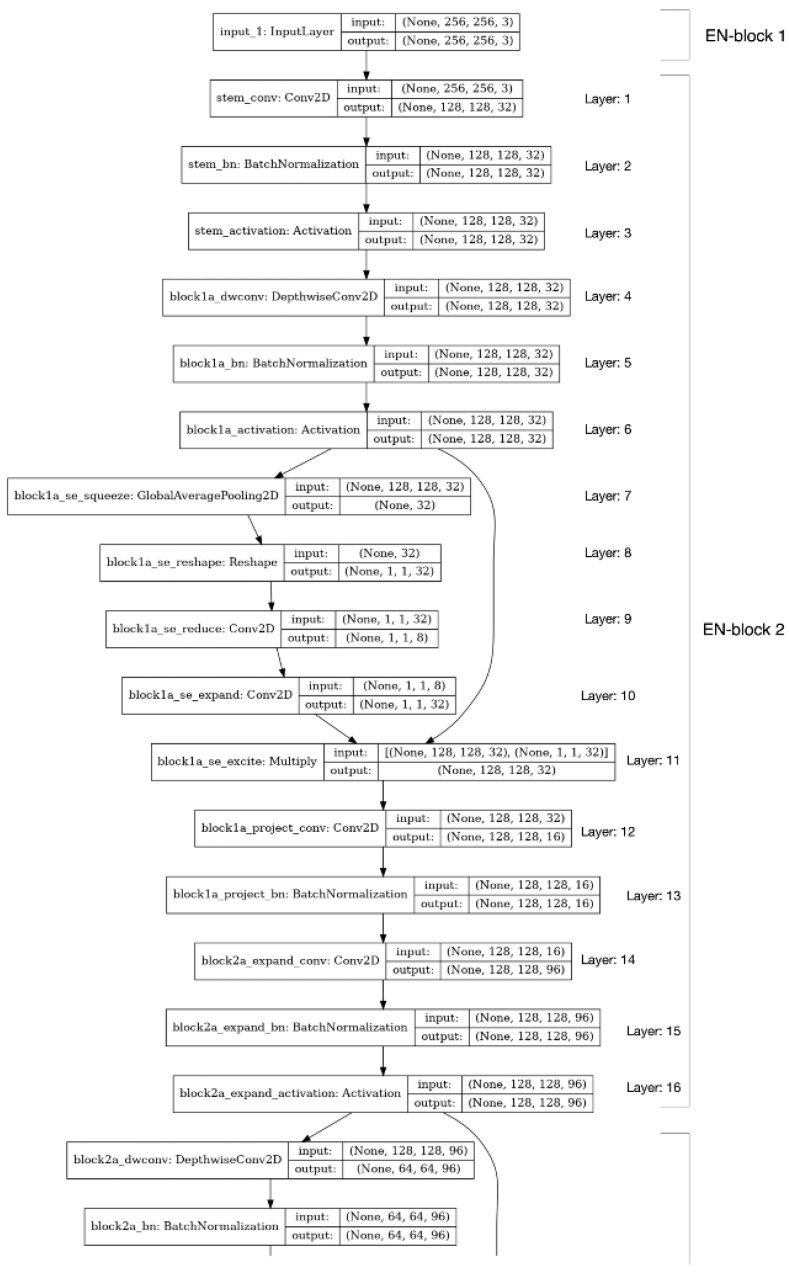
Layers in EN-block 1 and 2 of Rsef.

Figure 3 shows the detailed layers in EN-block 1 and 2 of Rsef. As shown in the figure, there are 16 layers in EN-block 2. Because layer 6 has a skip connection to layer 11 in EN-block 2, we also exclude layers from 6 to 10 as a split point. The final candidates for a feasible split point are layers 1, 2, 3, 4, 5, 11, 12, 13, 14, and 15. We generate possible partitioned models at each split point and benchmark the performance of each partitioned model by using an IoT device and the edge server. The optimal split point will be shown in the evaluation section.

During crack detection, Rsef-Edge in the IoT device receives an image as input and performs the early layers of Rsef before the split point. The output data is then transmitted to the edge server via the network. Rsef-Edge does not compress the output data during the transfer because compression may lead to a significant accuracy drop, which is critical in crack detection. Rsef-Edge in the edge server passes the received data to the remaining layers of Rsef on the edge server. After the remaining layers are executed with a GPU, the inference result is delivered to the IoT device.

## 5. Results

We evaluate the model accuracy and the inference time. The accuracy is to evaluate the best structure for the Rsef model, and the inference time is to investigate the speedup benefits of the Rsef-Edge.

### 5.1. Experimental Environment

We use a dataset—DAGM 2007 [31], which is a synthetic benchmark for defect detection of texture surface images. The DAGM 2007 dataset consists of the following 10 classes (or sub-datasets): 6 classes for development and 4 classes for competition at the 2007 symposium of the DAGM. Each of the first 6 classes has 1000 nondefective and 150 defective images, and each of the remaining 4 classes contains 2000 nondefective and 300 defective images. Each class is generated by a different texture and defect model. Defective images show one defect on the background texture. Weak labels are provided for denoting the defective area. The ratio of training data and testing data is set to 7:3 in the evaluation as with DenseNet [34]. We use the software and hardware for evaluating our model in Table 2. We run our model on two machines: the Rsef-Edge server and the Rsef-Edge IoT device. For the IoT device, we adopt the well-known Nvidia Jetson Tx2. For training, we use the Adam optimizer with a learning rate of 0.0005 and sum of Jaccard, and binary cross-entropy is used as a loss function. For validation set, we use the 15% of the original training set by splitting the set randomly.

### 5.2. Evaluation of Accuracy

The results in Table 3 are the evaluation of the segmentation prediction performance of the Rsef model. The metric is the average true positive rate (TPR or recall) and accuracy (ACC) computed as follows:

(3) $$TPR=\frac{TP}{TP+FN}$$

(4) $$ACC=\frac{TP+TN}{TP+TN+FP+FN}$$

where *TP*, *TN*, *FP*, and *FN* mean the number of true positives, true negatives, false positives, and false negatives, respectively.

In order to determine the final form of Rsef, various combinations of model elements of IDG, Albumentation (Albu), and Skip Connection (SC) were applied to the original model, which is an Encoder–Decoder model that consists of EfficientNet-B0 encoder and U-Net decoder without SC. The application target is 6 image classes belonging to the dataset, and the average true positive rates and average accuracy of all and top-5 classes were obtained. In the results, the overall performance showed the best prediction result when both SC and Albu were applied (Top 5 avg. Acc. 97.36%). Thus, we employ the original + SC + Albu model as Rsef.

We compare Rsef with DenseNet [34], which provides the accuracy results for each DAGM 2007 class 1 to 6. Except class 2, the avg. accuracy of DenseNet showed avg. 85.33% to 94.46% for four conditions. For the same classes, Rsef showed avg. 97.36% accuracy. However, the accuracy of class 2 is only 38.46%. We notice a similar result in DenseNet [34]. Class 2 is a dataset that cannot be accurately classified. The cause of this error is that the images in class 2 have small defects compared with other classes. For example, we observed that the actual pixel size of a defect in crack images in class 2 is small, but it has a relatively large crack region in label images. This makes training harder, and the proposed model cannot result in good performance.

### 5.3. Evaluation of Inference Time

**Figure 4:**
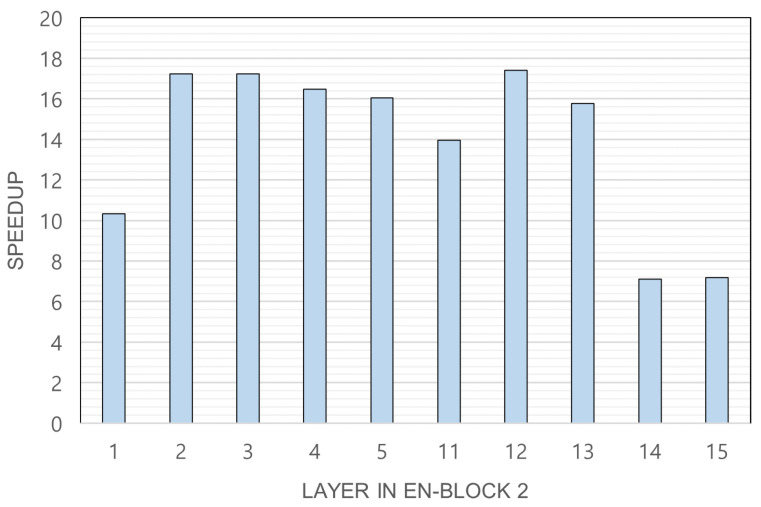
Latency speedup of Rsef-Edge. The speedup value is obtained by dividing the execution time of the IoT device by the time taken by Rsef-Edge.

As original + SC + Albu shows the best accuracy, we have measured the inference time using this model. As explained in Section 4.3, we have selected feasible split points as layers 1, 2, 3, 4, 5, 11, 12, 13, 14, and 15 in EN-block 2 and measured the inference time at each split point. Figure 4 shows the latency speedup of Rsef-Edge compared with the IoT device-only execution. The speedup is shown according to the feasible slicing point of Rsef. It is revealed that the best slicing point is layer 12, with a speedup of 17.40, but layers 2 and 3 are also good slicing points, with a speedup of 17.23 and 17.24, respectively. This result shows that Rsef-Edge is effective for crack detection in IoT devices that do not have powerful GPUs.

## 6. Conclusions

This paper suggests crack detection using edge computing. We develop Rsef, a machine-learning technique that receives images and quickly detects cracks. We also construct Rsef-Edge, an edge-based system to decrease the inference time of Rsef even in low-power IoT devices. We show both a fast inference time and a high level of accuracy in various test situations, including a low-powered computing environment. Thus, our suggestion is beneficial for live crack detection. Our evaluation results show that Rsef-Edge can mitigate the introduction cost and enhance the scalability by improving the inference time up to 17.40 times faster.

## Figures and Tables

**Table 1:** EN-Block structure of Rsef.

EN-Block No. Stage *i*	Operator $\hat{F_{i}}$	Channels $\hat{C_{i}}$	Resolution $\hat{H_{i}}\times \hat{W_{i}}$
-	Conv. 3 × 3	32	256 × 256
1	MBConv1. 3 × 3	16	128 × 128
2	MBConv6. 3 × 3 MBConv6. 3 × 3	24 24	128 × 128 128 × 128
3	MBConv6. 5 × 5 MBConv6. 5 × 5	40 40	64 × 64 64 × 64
4	MBConv6. 3 × 3 MBConv6. 3 × 3 MBConv6. 3 × 3 MBConv6. 3 × 3	80 80 80 80	32 × 32 32 × 32 32 × 32 32 × 32
5	MBConv6. 5 × 5 MBConv6. 5 × 5 MBConv6. 5 × 5	112 112 112	16 × 16 16 × 16 16 × 16
6	MBConv6. 5 × 5	192	16 × 16

**Table 2:** Experimental environment.

Item	Rsef-Edge Server	Rsef-Edge IoT Device (NVIDIA Jetson TX2)
CPU	Intel(R) Core (TM) i7-10700 CPU 2.90 GHz	ARM Cortex-A57 aarch64 2.03 GHz
GPU	GeForce RTX 3090 (single)	256-core NVIDIA Pascal (not used)
backbone	EfficientNet-B0	
optimizer	Adam (learning rate = 0.0005)	
image size	(256, 256, 3)	
tensorflow version	2.1.0	
python version	3.7.6	
keras version	2.3.1	

**Table 3:** Comparison of accuracy quality of Rsef candidate model. Avg. TPR and ACC indicate average true positive rates and accuracy for all classes, respectively. Top 5 avg. TPR and ACC indicates average TPR and accuracy for top five classes.

Model	Avg. TPR	Top-5 Avg. TPR	Avg. ACC	Top-5 Avg. ACC
original	89.33%	98.53%	84.07%	96.45%
original + IDG	92.51%	94.35%	91.86%	92.84%
original + Albu	88.70%	94.73%	87.56%	93.50%
original + SC	82.38%	98.85%	74.71%	89.77%
original + SC + IDG	93.00%	97.98%	89.50%	90.73%
original + SC + Albu	93.25%	98.99%	87.54%	97.36%

## Data Availability

Not applicable.
